# Clinical characterization of Idiopathic Restrictive Cardiomyopathy having rare variant (E949K) in β-cardiac myosin heavy chain gene

**DOI:** 10.1186/1755-8166-7-S1-P34

**Published:** 2014-01-21

**Authors:** Mitali Kapoor, Amitabh Biswas, Soumi Das, Sandeep Seth, Balram Bhargava, Vadlamudi Rao

**Affiliations:** 1 Department of Anthropology, University of Delhi, Delhi, India; 2 Department of Cardiology, AIIMS, India

**Keywords:** MYH7 gene, Cardiac catheterization, phenotypic heterogeneity

## Background

Idiopathic Restrictive cardiomyopathy (IRCM) and hypertrophic cardiomyopathy (HCM) reflects the same or very similar disorders showing restrictive physiology with different names due to discretionary crosscuts in the LV wall thickness rather than two separate distinct diseases. The perspective of this study is to clinically evaluate the IRCM proband and comparison between the two distinct disease phenotype IRCM and HCM as an outcome of same genotype i.e.E949K in gene MYH7.

## Methods

Diagnosis was based on ECG, 2D-echocardiography, Cardiac Catheterisation and Endomyocardial biopsy. 5ml of Blood sample was collected and DNA was isolated using phenol-chloroform method. Sequencing of the hotspot region of MYH7 gene i.e. exon 23 by Sanger method (ABI 3730) was done. Screening of family members available was also done clinically as well as genetically. The study was ethically approved by Institutional committee and informed written consent was taken from all participants.

## Results

The proband, 11 year old male reported with chest discomfort, syncope, palpitations and arrthymias since last six months. Echocardiography showed marked biatrial enlargement (LA=46mm, RA=54mm), normal sized ventricles (IVS=8mm, LV=9mm) with mild MR and severe TR. Cardiac catheterization revealed grade III MR, severe TR severe PAH, mild RV systolic dysfunction. With these evidences a diagnosis of Idiopathic RCM was made.

Sequencing of exon 23 of MYH7 gene led to the identification of rare variant E949K (c.2845G>A) in MYH7 gene. This variant was not found in our screening of 80 cardiomyopathies patients and 78 unaffected family members. This mutation was previously reported by Watkins et al.1992 in a case of familial HCM and suggested late onset of disease with severe LV hypertrophy and Rayment I et al.1995 also reported that this mutation shows loss of tensile strength of stiffness which represents the main phenotype of IRCM. We found this rare mutation in IRCM patient with early onset and no ventricular hypertrophy.

## Conclusion

This study represents the phenotypic heterogeneity of same rare variant in two ethnically different probands indicating environmental role or mutations in some other genes. This study reiterates the importance of whole genome sequencing approaches in clinical practice.

